# Supplementary material for: A fault-intrusion-tolerant system and deadline-aware algorithm for scheduling scientific workflow in the cloud
Source: PeerJ Comput Sci. 2021 Nov 2;7:e747. doi: 10.7717/peerj-cs.747 (PMC8576554; doi:10.7717/peerj-cs.747)
Supplement: Supplemental Information 6 [file peerj-cs-07-747-s006.zip › Myproject1.1/docs/org/workflowsim/clustering/class-use/ClusteringInterface.html]

Uses of Interface org.workflowsim.clustering.ClusteringInterface


JavaScript is disabled on your browser.


- Overview
- Package
- Class
- Use
- Tree
- Deprecated
- Index
- Help

- Prev
- Next

- Frames
- No Frames

- All Classes

## Uses of Interface org.workflowsim.clustering.ClusteringInterface

- Packages that use ClusteringInterface

  | Package | Description |
  | --- | --- |
  |  |  |
  | --- | --- |
  | org.workflowsim.clustering |  |
  | org.workflowsim.clustering.balancing |  |
- - ### Uses of ClusteringInterface in org.workflowsim.clustering

    Classes in org.workflowsim.clustering that implement ClusteringInterface

    | Modifier and Type | Class and Description |
    | --- | --- |
    |  |  |
    | --- | --- |
    | `class` | `BasicClustering` The default clustering does no clustering at all, just map a task to a job |
    | `class` | `BlockClustering` BlockClustering groups tasks in both horizontal and vertical direction |
    | `class` | `HorizontalClustering` HorizontalClustering merges task at the same horizontal level |
    | `class` | `VerticalClustering` VerticalClustering merges tasks at the same pipeline |
  - ### Uses of ClusteringInterface in org.workflowsim.clustering.balancing

    Classes in org.workflowsim.clustering.balancing that implement ClusteringInterface

    | Modifier and Type | Class and Description |
    | --- | --- |
    |  |  |
    | --- | --- |
    | `class` | `BalancedClustering` BalancedClustering is a clustering method that aims balancing task runtime and data dependency. |

- Overview
- Package
- Class
- Use
- Tree
- Deprecated
- Index
- Help

- Prev
- Next

- Frames
- No Frames

- All Classes
